# Modeling and Predicting Tissue Movement and Deformation for High Intensity Focused Ultrasound Therapy

**DOI:** 10.1371/journal.pone.0127873

**Published:** 2015-05-20

**Authors:** Xiangyun Liao, Zhiyong Yuan, Qianfeng Lai, Jiaxiang Guo, Qi Zheng, Sijiao Yu, Qianqian Tong, Weixin Si, Mingui Sun

**Affiliations:** 1 School of Computer, Wuhan University, Wuhan, Hubei, China; 2 Department of Computer Science and Engineering, The Chinese University of Hong Kong, Hong Kong, China; 3 School of Medicine, University of Pittsburgh, Pittsburgh, Pennsylvania, United States of America; University of Minnesota, UNITED STATES

## Abstract

**Purpose:**

In ultrasound-guided High Intensity Focused Ultrasound (HIFU) therapy, the target tissue (such as a tumor) often moves and/or deforms in response to an external force. This problem creates difficulties in treating patients and can lead to the destruction of normal tissue. In order to solve this problem, we present a novel method to model and predict the movement and deformation of the target tissue during ultrasound-guided HIFU therapy.

**Methods:**

Our method computationally predicts the position of the target tissue under external force. This prediction allows appropriate adjustments in the focal region during the application of HIFU so that the treatment head is kept aligned with the diseased tissue through the course of therapy. To accomplish this goal, we utilize the cow tissue as the experimental target tissue to collect spatial sequences of ultrasound images using the HIFU equipment. A Geodesic Localized Chan-Vese (GLCV) model is developed to segment the target tissue images. A 3D target tissue model is built based on the segmented results. A versatile particle framework is constructed based on Smoothed Particle Hydrodynamics (SPH) to model the movement and deformation of the target tissue. Further, an iterative parameter estimation algorithm is utilized to determine the essential parameters of the versatile particle framework. Finally, the versatile particle framework with the determined parameters is used to estimate the movement and deformation of the target tissue.

**Results:**

To validate our method, we compare the predicted contours with the ground truth contours. We found that the lowest, highest and average Dice Similarity Coefficient (DSC) values between predicted and ground truth contours were, respectively, 0.9615, 0.9770 and 0.9697.

**Conclusion:**

Our experimental result indicates that the proposed method can effectively predict the dynamic contours of the moving and deforming tissue during ultrasound-guided HIFU therapy.

## Introduction

High Intensity Focused Ultrasound (HIFU)[1–3] therapy capitalizes on two properties of ultrasound, tissue penetration and deposition, by externally focusing an ultrasound beam on diseased or damaged tissue (the therapeutic target). Thus, through mechanical, thermal and cavitation effects, HIFU performs treatment by heat-ablating the target tissue using precisely localized high-intensity energy. Because of the safety and efficacy [4–9], HIFU has been increasingly applied to the treatment of cancerous growths, such as uterine fibroids, breast fibroadenoma, hepatocellular carcinoma (HCC), osteosarcoma, and prostate cancer [10–15]. Recently, Tatiana D *et al*. demonstrated the feasibility of boiling histotripsy to HIFU-induced ultrasound-guided tissue fractionation in an *in vivo* pig model, allowing treatment of tissue immediately adjacent to major blood vessels and other connective tissue structures [16]. However, the target tissue such as a tumor often moves and/or deforms during ultrasound-guided HIFU therapy because of the existence of an external force. As a result, HIFU may appear to be targeting diseased tissue when in fact it is impinging on healthy tissue, leading to serious complications [17]. To avoid this problem, the general practice during surgery is that experienced doctors manually target the HIFU treatment head to a safe area (not 100% of the disease tissue) by simply observing the movement and deformation characteristics of the target tissue. However, this practice causes a portion of the lesion to survive from the treatment, potentially leading to tumor recurrence after surgery. Furthermore, the need to continuously relocate the target tissue as it moves and deforms can substantially increase the time of surgery, which causes additional pain for the patient and increase in treatment cost. If we can dynamically and accurately predict the motion and deformation of the target tissue and make timely adjustments to the positioning of the HIFU target, the risk of surgery (including both surgical and recurrent risks) can be reduced substantially. Despite the importance, there has been a lack of research on this important problem. This work aims to find an effective solution that improves both safety and outcome of the ultrasound-guided HIFU surgery.

We propose the use of computational dynamic modeling and prediction of tissue motion/deformation during HIFU therapy. By predicting the position of target tissue under an external force, we can adjust the focal region during HIFU therapy accordingly so that the HIFU treatment head remains aligned with the target tissue. To accomplish this goal, we first collect a spatial sequence of scanned ultrasound images of the target tissue. A method based in the Geodesic Localized Chan-Vese (GLCV) modes developed to segment the target tissue. We then construct a 3D model based on our segmentation results, apply the external force to it, and propose a versatile particle framework to model the experimental environment. Our method allows us to compute the movement and deformation of the target tissue as it responds to the external force.

To reconstruct a 3D model of the target tissue, we must obtain a static contour of the target tissue. Traditionally, during ultrasound-guided HIFU therapy, doctors manually mark the target tissue’s contour; however, manual identification of the contour is time-consuming and greatly affects the surgical process [18]. Therefore, a significant need exists to develop faster methods for defining target tissue’s contour in multiple sections. The method most commonly used for this task is contour segmentation, which partitions an image into multiple segments and extracts the interested target. Contour segmentation is typically used to locate objects and boundaries in images. As HIFU ultrasound images usually have a low Signal-to-Noise Ratio (SNR), weak borders and uneven grey level distribution, A precise segmentation of these images has proven to be difficult. Caselles *et al*.[19] proposed a Geodesic Active Contour (GAC) model based on curve evolution theory and the level set method. This model uses gradient information as an image-based “force” to push the curve towards the target edge and produces good segmentation results for targets that have clearly defined edges. However, as the gradient represents highly localized information within a given image, this method is sensitive to noise and may lead to apparent edge leakage in low contrast images. Chan and Vese [20] proposed the Chan-Vese (CV) model, which can effectively segment noisy and low contrast images as it does not rely on the gradient information. However, this method can lead to an incorrect interpretation of non-evenly distributed images, as the model assumes that regions within the image are evenly distributed and uses global statistical information. To segment uneven images, Li *et al*.[21] introduced a kernel function to define the local binary fitting energy in a variational level set framework, embedding local grey level information into the model. Lankton *et al*.[22] proposed a localized CV (LCV) model that allows a region-based energy formula to be rewritten into a localized form. Li *et al*.[23] proposed a Region-Scalable Fitting (RSF) model where a kernel function in the data fitting term was provided to define local binary fitting energy, but it causes fragmentation in segmentation when images have severe intensity inhomogeneity. The primary shortcoming of the localized region-based active contour models discussed above is that they are sensitive to initial contours. Recently, Liao *et al*.[24] presented a multi-scale and shape constrained localized CV model (MSLCV) to segment uterine fibroid ultrasound images in HIFU therapy and achieved good segmentation results. It adopts a multi-scale segmentation method to improve segmentation efficiency. However, the final segmentation largely depends on segmentation of images in coarse-scales and this model is specially designed for uterine fibroid ultrasound images. Combining GAC and LCV, we provide a geodesic LCV (GLCV) model to overcome the above shortcomings. Through the analysis of the uniformity of grey level distribution around each point on the evolution curve, our model weighs the contributions of each model during image processing. Therefore, our method not only segments the target tissue accurately and effectively, but also increases the efficiency of ultrasound-guided HIFU therapy by eliminating the procedure of manual segmentation.

There are various types of material that makes up the surgical environment during the ultrasound-guided HIFU therapy, including elastic tissue, rigid body and intermediates such as fluids. The target tissue moves and deforms because of the existence of external forces. Thus, predicting the movement and deformation of the target tissue is actually a multiphase coupling process among elastic tissues, rigid body and fluids intermediates. In recent years, numerous methods have been proposed to physically model the properties of fluids and solids. These methods generally can be classified generally as either mesh-based methods, such as Finite Element Method (FEM) or meshless methods. The Smoothed Particle Hydrodynamics (SPH) has become a widely used meshless method to model fluids [25]. Its particle properties make it also suitable for modeling deformable objects and fluids [26, 27]. Many state-of-the-art methods are proposed to calculate the fluid-solid coupling such as rigid bodies and deformable solids [28–30]. Although excellent results have been achieved in modeling rigid bodies, fluids and deformable solids, the coupling of different materials involves a high computational cost. For example, when coupling two different materials, such as SPH fluid and FEM solid, addressing data exchange becomes a difficult task. As the complexity of simulation scenarios increases, there is a strong need for the development of a flexible, accurate and versatile multiphase coupling method applicable to complex models and various virtual simulation scenarios. In this work, we propose a novel, versatile particle framework that can accurately simulate the multiphase coupling of elastic tissue, rigid body and fluids.

To quantitatively verify our method, we utilized a sample of cow tissue as an experimental target tissue, which is suitable for simulating tumors such as uterine fibroids. We first placed the target tissue in a water pool of ultrasound-guided HIFU equipment and then used a rigid body (metal bar) to applied forces, leading to its movement and deformation. Using the ultrasound-guided HIFU equipment, we first obtained the spatial sequence of scanned ultrasound images of the target tissue. Then, we recorded both mechanical movement and deformation of one cross-section of the target tissue under the external force. Our experimental data verifies that our method can effectively predict the movement and deformation of the target tissue. The main contributions of this paper include:

- The demonstration that HIFU ultrasound image segmentation is capable of obtaining the contours of the target tissue accurately and effectively, which may replace the current manual operation and increase the efficiency of the ultrasound-guided HIFU therapy.
- The development of a versatile particle framework and an iterative parameter estimation method, which allows multi-phase coupling among target tissue, rigid body and fluids.
- The creation of a new method to predict the movement and deformation of target tissue during ultrasound-guided HIFU therapy.

## Materials and Methods

### 2.1 HIFU Ultrasound Images Segmentation

We utilized a sample of cow tissue as our experimental target tissue, which was purchased at Carrefour near Chongqing Medical University, Yuzhong District, Chongqing, China. All HIFU ultrasound images of the target tissue were acquired by using the HIFU equipment of the Model JC200 Focused Ultrasound Tumor Therapeutic System [31]. In the following, we will first describe the localized CV (LCV) model and then propose our geodesic LCV (GLCV) model to segment the HIFU ultrasound images.

#### 2.1.1 LCV model

To overcome the difficulties in processing images with non-evenly distributed intensity levels, Lankton *et al*.[22] proposed the LCV model which allows any global region-based energy formula to be rewritten into localized form. To define a localized region for each point on the curve, a characteristic function is defined as:

$$B\text{(}x,y\text{)}=\left\{ \begin{array}{l} 1,\;\left\|x-y\right\|<r\\ 0,\;otherwise. \end{array}\right.\;$$ (1)

where *x*, *y* ∈ Ω represents each point and *r* represents the radius parameter.

The average intensities, *c*_*x*1_ and *c*_*x*2_, inside and outside of the local region of point *x* on the contour are defined by the characteristic function *B*(*x*, *y*) [22]:

$$c_{x1}=\frac{\int_{\Omega_{y}}B(x,y)\cdot H(\phi (y))\cdot I(y)dy}{\int_{\Omega_{y}}B(x,y)\cdot H(\phi (y))dy}$$ (2)

$$c_{x2}=\frac{\int_{\Omega_{y}}B(x,y)\cdot (1-H(\phi (y)))\cdot I(y)dy}{\int_{\Omega_{y}}B(x,y)\cdot (1-H(\phi (y)))dy}$$ (3)

where *ϕ*(*y*) and *H*(*ϕ*) represent, respectively, the level set function and the Heaviside function, Ω_*y*_ is the local region defined by *B*(*x*, *y*), and *I*(*y*) is the intensity of point *y*. The energy function of LCV model is given by:

$$E^{\text{LCV}}\left(c_{x1},c_{x2},\phi \right)=\int_{\Omega_{x}}\delta (\phi (x))\int_{\Omega_{y}}B(x,y)\cdot F_{region}(I(y),\phi (y))dydx+\mu \int_{\Omega_{x}}\delta (\phi (x))\left\|\nabla \phi (x)\right\|dx$$ (4)

Where Ω_*x*_ and Ω_*y*_ are the integration domain, F_region_ = *H*(*ϕ*(*y*)) (*I*(*y*)-*c*_*x*1_)^2^+(1-*H*(*ϕ*(*y*))) (*I*(*y*)-*c*_*x*2_)^2^ represents the region-based force, *δ*(*ϕ*) represents the Dirac function, *x* is a global point within the image, and *y* is a local point within the local region of the circle centered at *x*. The curvature flow for point *x* is defined as:

$$\frac{\partial \phi }{\partial t}(x)=\delta (\phi (x))[\int_{\Omega_{y}}B(x,y)\delta (\phi (y))\cdot ({\left(I(y)-c_{x1}\right)}^{2}-{\left(I(y)-c_{x2}\right)}^{2})dy+\mu div(\frac{\nabla \phi (x)}{\left|\nabla \phi (x)\right|})]$$ (5)

In the localized version, the energy becomes minimized when every point on the curve has moved such that the localized inside and outside become the best estimates of the averages of *c*_*x*1_ and *c*_*x*2_ in local region.

#### 2.1.2 GLCV model

The LCV model is effective in segmenting images with non-evenly distributed intensity. However, due to its localized characteristics, the LCV model is sensitive in defining an initial contour. On the other hand, the Geodesic Active Contour (GAC)[19] model can easily capture image edge information and has the advantage of fast convergence. An effective strategy would thus be the development of a new model with desirable features of both LCV and GAC models. We call the new model the geodesic LCV (GLCV) model.

In regions with low contrast or nearly uniform distribution in ultrasound images, the force values generated by LCV model are small, resulting in a slow curve evolution. Therefore, we aim to increase the force based on the edge gradient information in these regions to accelerate curve evolution. To accomplish this, we evaluate the uniformity of grey level distribution around each image point on the evolution curve to decide how much weight to give the GAC model when combining it with the LCV model. A characteristic function *t*(*x*) is designed to measure the grey level uniformity around each point.

$$t\text{(}x\text{)}=\left\{ \begin{array}{l} 1,\;\left|c_{x1}-c_{x2}\right|<\kappa_{1}\\ 0,\;otherwise. \end{array}\right.\;$$ (6)

where *c*_*x*1_ and *c*_*x*2_ were defined in (2) and (3). When the absolute value of the difference between *c*_*x*1_ and *c*_*x*2_ is less than a threshold *κ*_1_, we consider the region surrounding point *x* to be of low contrast and approximately uniform, and introduce the force based on GAC model’s edge gradient information to accelerate curve evolution. The level set evolution equation of GLCV model can be defined as:

$$\begin{array}{l} \frac{\partial \phi }{\partial t}(x)=\delta (\phi (x))[\int_{\Omega_{y}}B(x,y)\delta (\phi (y))\cdot ({\left(I(y)-c_{x1}\right)}^{2}-{\left(I(y)-c_{x2}\right)}^{2})dy+\mu div(\frac{\nabla \phi (x)}{\left|\nabla \phi (x)\right|})]\\ \;+t(x)\;\delta (\phi (x))\left[g\left|\nabla \phi (x)\right|div(\frac{\nabla \phi (x)}{\left|\nabla \phi (x)\right|})\;+\;\nabla g\cdot \nabla \phi \;+\;\alpha \;g\left|\nabla \phi (x)\right|\right] \end{array}$$ (7)

where the first and second items represent, respectively, local region fitting and geodesic flow of edge detection, *α* is a constant, and *g* represents the edge detection function defined as: *g* = 1/(1+|∇*G*_*σ*_(*x*) *I*(*x*)|^2^) with *G*_*σ*_ being the Gaussian kernel function with standard deviation *σ*.

The GLCV model simultaneously makes use of both local regional information and edge information in regions with low contrast or nearly uniform distribution. Thus, this model helps accelerate curve evolution and prevents boundary leakage. However, when the curve is near the target edge, the value of *t*(*x*) is 0 and the LCV model dominates which segments the target accurately.

### 2.2 Dynamic Modeling and Prediction

To model and predict the movement and deformation of the target tissue, a 3D tissue model is developed. We first segment the spatial sequence of scanned ultrasound images of the target tissue using the GLCV model. Then, we adopt a 3D regional growth reconstruction algorithm [32] to reconstruct a 3D model of the target tissue.

**Fig 1:**
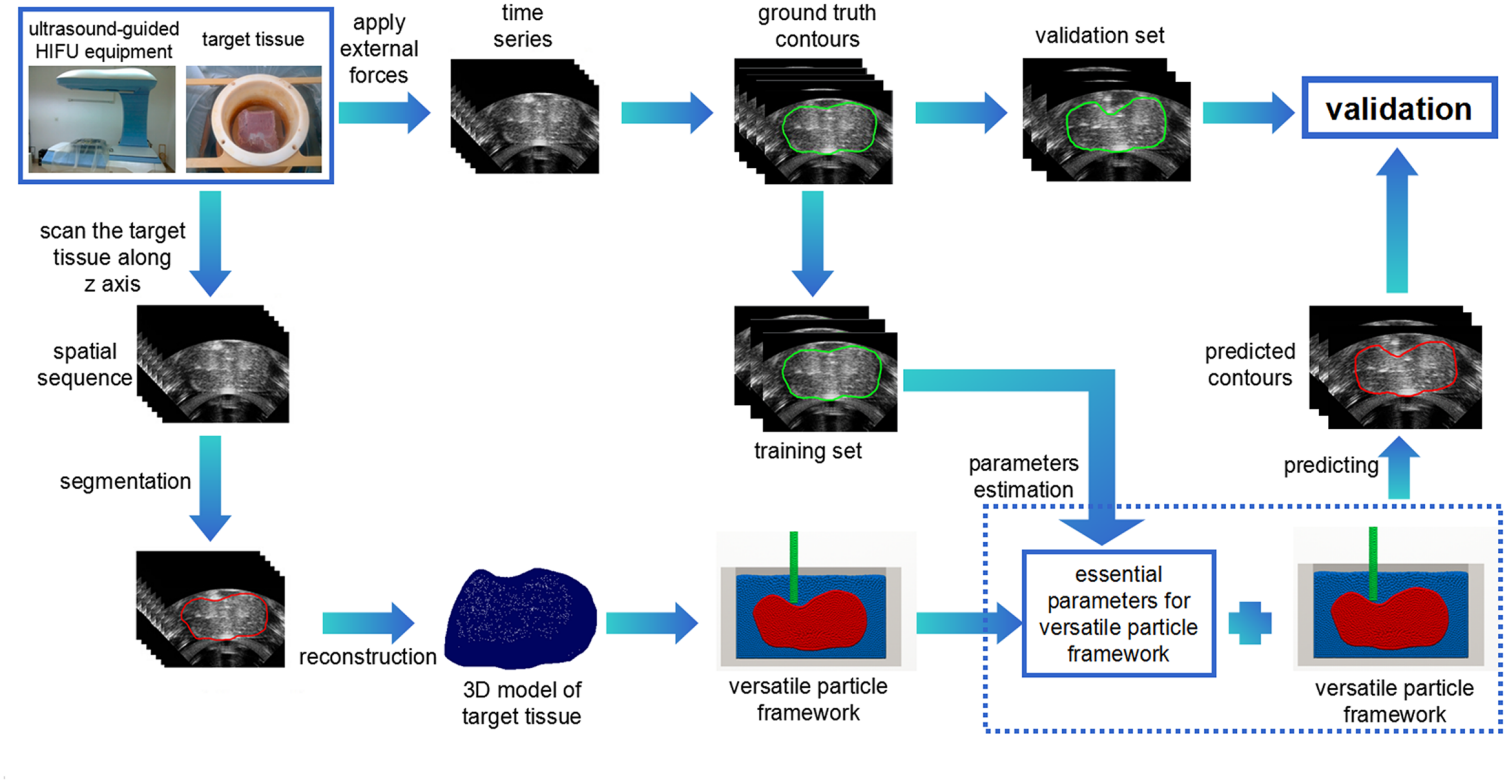
Process of dynamic modeling and prediction of target tissue movement and deformation: The target tissue is placed in the water pool of the ultrasound-guided HIFU equipment. First, we scan ultrasound images of spatial sequence of the target tissue and use the GLCV model to obtain the segmentation results, and then reconstruct a 3D model of the target tissue. Second, we apply the external force to the target tissue using a rigid body while the ultrasound-guided HIFU equipment records time series of ultrasound images of the target tissue’s one cross section. The segmentation results are manually segmented by an experienced doctor and as the ground truth which is divided into two parts: training set and validation set. Third, we propose a versatile particle framework to model the movement and deformation of target tissue. Combined with the versatile particle framework, the external force and other essential parameters are acquired through the iterative parameter estimation algorithm. By applying these parameters to the versatile particle framework, we predict the target tissue’s subsequent movement and deformation, which is used to compare with the validation set to verify the effectiveness of our method.

In our study, the target tissue was placed in a water pool of the ultrasound-guided HIFU equipment. We then applied a force to the target tissue using a rigid body while the ultrasound-guided HIFU equipment recorded time series of ultrasound images of the target tissue’s one cross section under the external force. To model this process, a versatile particle framework was created for the 3D model of the target tissue and its experimental environment to calculate and predict its movement and deformation. Part of the ultrasound images of time series were utilized to estimate the necessary parameters of the versatile particle framework, while another part of ultrasound images of time series were used for the validation of the predicted contours of the target tissue. The process of our method is illustrated in Fig 1.

#### 2.2.1 versatile particle framework

In this section, the versatile particle framework is described for target tissue modeling, rigid body modeling, and fluid modeling and coupling.

**SPH fluids:** The Navier-Stokes equations [33] are used to describe fluidic motions where the momentum equation used in the equations can be written in the form of Newton's second law as follows:

$$\rho \frac{dv}{dt}=-\nabla p+\mu \nabla^{2}v+f$$ (8)

where *ρ* is the fluid density, **v** is velocity, *p* is pressure, and *μ* is the viscosity coefficient. The terms on the right side represent pressure, viscous force and external force.

We adopted the SPH [28] method to solve Navier-Stokes equations. The basic idea of the SPH method is to describe a continuum for fluids or solids by using interactive particle groups. According to SPH, a scalar quantity *A*(**r**) is interpolated at location **r** by a weighted sum of contributions from all particles:

$$A\left(r\right)=\sum_{j}A_{j}\frac{m_{j}}{\rho_{j}}W\left(r-r_{j},h\right)$$ (9)

where *W* is smooth kernel function which can be the cubic spine polynomial, **r**_*j*_ is the particles within distance *h* from particle **r**, *A*_*j*_ represents the quantity of particle **r**_*j*_, and *m*_*j*_ and *ρ*_*j*_ are, respectively, mass and density. By replacing *A*_*j*_ with density, the density can be obtained as:

$$\rho \left(r_{i}\right)=\sum_{j}\rho_{j}\frac{m_{j}}{\rho_{j}}W\left(r_{i}-r_{j},h\right)=\sum_{j}m_{j}W\left(r_{i}-r_{j},h\right)$$ (10)

Similarly, we can calculate the pressure of each particle by replacing *A*_*j*_ with pressure intensity. To obtain a homogeneous symmetric pressure, we adopt the arithmetic mean of the pressure intensities of particles **r** and **r**_*j*_ as the particle’s pressure intensity:

$$f_{{}_{i}}^{pressure}{}_{}=\sum_{j}\frac{m_{j}\left(p_{i}+p_{j}\right)}{2\rho_{j}}\nabla W\left(r_{i}-r_{j},h\right)$$ (11)

where *p*_*i*_ = *K*(*ρ*_*i*_-*ρ*_0_), *ρ*_0_ represents the initial fluid density, and *K* is a constant related to the fluid. The viscosity force is defined as:

$$f_{i}^{\text{viscosity}}=\sum_{j}\mu \left(v_{j}-v_{i}\right)\frac{m_{j}}{\rho_{j}}\nabla^{2}W\left(r_{i}-r_{j},h\right)$$ (12)

The initial external force for a single particle is the gravity. We can calculate the above force using Newton’s second law to determine the acceleration and new position for each particle within our system.

**Elastic tissue:** The elastic force generated by particle *i* is **f**_i-elastic_ = -∇**u**_*i*_ *U*, where **u**_*i*_ is particle’s displacement. Here the strain energy is *U* = ε·**σ**/2. We utilize stress-strain relationship: **σ = Cε**, where **C** is a four-order tensor computed by the Young’s modulus *E* and Poisson’s ratio *η*. The position is initialized to **x**, and the new position becomes **x**+**u** after the displacement of **u**. The Jacobian of this mapping is given by $J=I+\nabla u^{T}={\left[\begin{array}{ccc} J_{1}^{T} & J_{2}^{T} & J_{3}^{T} \end{array}\right]}^{T}$. We compute strain **ε** based on the quadratic Green-Saint-Venant strain tensor [34]:

$$\varepsilon =J^{T}J-I=\nabla u+\nabla u^{T}+\nabla u\nabla u^{T}$$ (13)

We compute derivatives ∇**u** using a moving least squares approximation [34]. The elastic force exerted on particle *i* is computed by the formula:

$$f_{\text{i-elastic}}=-2v_{i}J_{i}\sigma_{i}{\text{(}\sum_{\text{j}}x_{ij}x_{ij}^{T}w_{ij})}^{-1}\left(-\sum_{j}{\text{x}}_{ij}w_{ij}\right)$$ (14)

where *v*_*i*_ represents the volume of particle *i*, *v*_*i*_ = *m*_*i*_/*ρ*_*i*_, and *w*_*ij*_ = *W*(‖**x**_*j*_-**x**_*i*_‖, *h*_*i*_)is a kernel function.

The volume inverting displacement field can also contribute to elastic deformation. Hence, we present the volume conservation energy *U*_*v*_ = *k*_*v*_(‖**J**‖-1)^2^/2. The volume conservation force is the negative gradient of the volume conservation energy:

$$f_{\text{i-volume}}=-\nabla u_{i}U_{v}=-k_{v}(\left\|J\right\|-1)\nabla u_{i}\left\|J\right\|$$ (15)

After discretizing by SPH, the volume conservation force [26] becomes:

$$f_{\text{i-volume}}=-v_{i}k_{v}\left(\left\|J\right\|\text{-}1\right){\left(\sum_{\text{j}}x_{ij}x_{ij}^{T}w_{ij}\right)}^{-1}\left(-\sum_{j}x_{ij}w_{ij}\right)\left[\begin{array}{c} {\left(J_{2}\times J_{3}\right)}^{T}\\ {\left(J_{3}\times J_{1}\right)}^{T}\\ {\left(J_{1}\times J_{2}\right)}^{T} \end{array}\right]$$ (16)

Elastic tissue has the rheological properties such as stress relaxation, creep, hysteresis, etc. Therefore, we introduce viscosity forces as described in the Navier-Stokes equations when generating a particle-based elastic tissue model to describe its rheological properties. The viscosity forces between elastic tissue particles are computed by Eq (12). Here $\mu_{j↔i}=\beta_{1}{\left(\sqrt{1\text{-}\cos^{2}\theta }\left\|v_{i}-v_{j}\right\|/\left\|r_{i}-r_{j}\right\|\right)}^{\beta_{2}}$ defines the viscosity coefficient [35], where *β*_1_ *and β*_2_ are variables associated with the rheological body, and *θ* is the angle between **v**_*i*_-**v**_*j*_ and **r**_*i*_-**r**_*j*_.

**Rigid body:** The types of motion that a rigid body can adopt include translation and rotation. The external forces of rigid body particles include gravity and the coupling forces between fluids and solids. The resultant force of a rigid body is $F_{rigid}^{total}=\sum_{i=1}^{n_{r}}f_{i}$, where *n*_*r*_ represents the number of rigid body particles and **f**_*i*_ is the external force of rigid body particle *i*. The rigid body rotates according to

$$\tau_{rigid}^{total}=\sum_{i=1}^{n_{r}}\left(x_{i}-x_{r}\right)\times f_{i}.$$ (17)

**Multiphase coupling:** During the coupling process, particles of fluid, elastic tissue, and rigid body interact with each other. Particles are influenced by a coupling force, which impedes their movement. Taking elastic tissue particle models and fluid particle models as examples [26], we can calculate the coupling force **f**_*f*↔*e*_ by

$$f_{f↔e}=-K_{c}m_{f}m_{e}\nabla W_{}$$ (18)

where *K*_*c*_ is the coupling coefficient, *m*_*f*_ and *m*_*e*_ are, respectively, the masses of bodily fluid particles and elastic tissue particles, and *W* is the kernel which can be the cubic spine polynomial. We adopt the principle of momentum conservation to calculate the collision response

$$m_{f}v_{f-next}=m_{f}v_{f}-f_{f↔e}\cdot \Delta t$$ (19)

$$m_{e}v_{e-next}=m_{e}v_{e}-f_{f↔e}\cdot \Delta t$$ (20)

where **v**_*f*-*next*_ and **v**_*e*-*next*_ are, respectively, the velocities of fluid particles and soft tissue particles.

#### 2.2.2 Iterative parameter estimation

To model the movement and deformation of target tissue, it is necessary to acquire the essential parameters of the versatile particle framework (external force, Young modulus, Poisson’s ratio, etc.) of the versatile particle framework. The external force as well as the other essential parameters is scarcely measurable in the experiment. Obviously, it is inapplicable to use the traditional method to predict the target tissue’s motion, which requires all priori essential parameters.

We have acquired the ultrasound images of time series of the mechanical movement and deformation of the target tissue at one cross section under the external force. We utilize part of the time series of ultrasound images and adopt their ground truth contours as the training set and propose an iterative parameter estimation method to acquire the essential parameters of the versatile particle framework, making our method applicable without the priori essential parameters.

As the target tissue’s motion is relatively small and slow and the deformation time is relatively short, we assume the external forces as the average external force. Assuming that the estimated parameter set is *K*. For the training data, the particles’ movement and deformation set (measured as displacement) around the force bearing particle is *D*_*t*_ at time *t*, *t* = 1, 2, …, *n*. Assuming that the set of contour points utilized to estimate *K* at time *t* is *A*, and *A* = (*a*_1_, *a*_2_,…, *a*_*t*_). We set the following rules:

1. At time *t*, calculating ${\hat{D}}_{t}$ based on the estimated parameter ${\hat{K}}_{t-1}$. Assuming that *S*_*t*_ is the sum of the absolute value of the difference between ${\hat{D}}_{t}$ and D_t_ for all elements in *A*. *T* is defined as the threshold. Accepting ${\hat{K}}_{t-1}$ if S_i_≤T at time i(i = 1,2,…,t), that means ${\hat{K}}_{t}={\hat{K}}_{t-1}$.
2. If there exists S_i_>T for a_i_(a_i_∈A), making adjustments to ${\hat{K}}_{t-1}$ until there exists S_i_≤T for all the elements *a*_*i*_ in *A*.

The iterative parameter estimation algorithm is described in Table 1.

**Table 1:** Iterative Parameter Estimation Algorithm.

Step 1: At time *t* = 1, initializing ${\hat{K}}_{1}$ which makes *S*_1_≤*T*/2.
Step 2: At time *t* = *t*+1, calculating the movement and deformation based on parameter set ${\hat{K}}_{t-1}$. Assign ${\hat{K}}_{t}={\hat{K}}_{t-1}$ if it conforms to the rule (1). Otherwise, making adjustments according to rule (2) and obtaining ${\hat{K}}_{t}$.
Step 3: Repeating step 2 until ${\hat{K}}_{n}$ satisfies *S*_*t*_≤*T*, *t* = 1,2,…, *n*. Then ${\hat{K}}_{n}$ is the parameter set.

The advantages of the iterative parameter estimation algorithm are:

1. At time *t* = 1, adjustment to *K*_1_ subject to *S*_1_≤*T*/2 helps reduce the time of adjustments of ${\hat{K}}_{t-1}$ for *t*>1.
2. The algorithm estimates parameter set *K* by using the step iterative method. It is easy to make adjustments to ${\hat{K}}_{t-1}$ if the number of elements in *A* is small.
3. The amount of calculation is relatively small.

## Results

The experimental platform includes: (1) hardware: Intel Xeon E3-1230 CPU, 3.30GHz, 2GB memory, Geforce GTX 650Ti; (2) software: Visual Studio 2010, Matlab R2011a, Visual Studio 2012, OpenGL.

### 3.1 HIFU Ultrasound Image Segmentation Results

**Fig 2:**
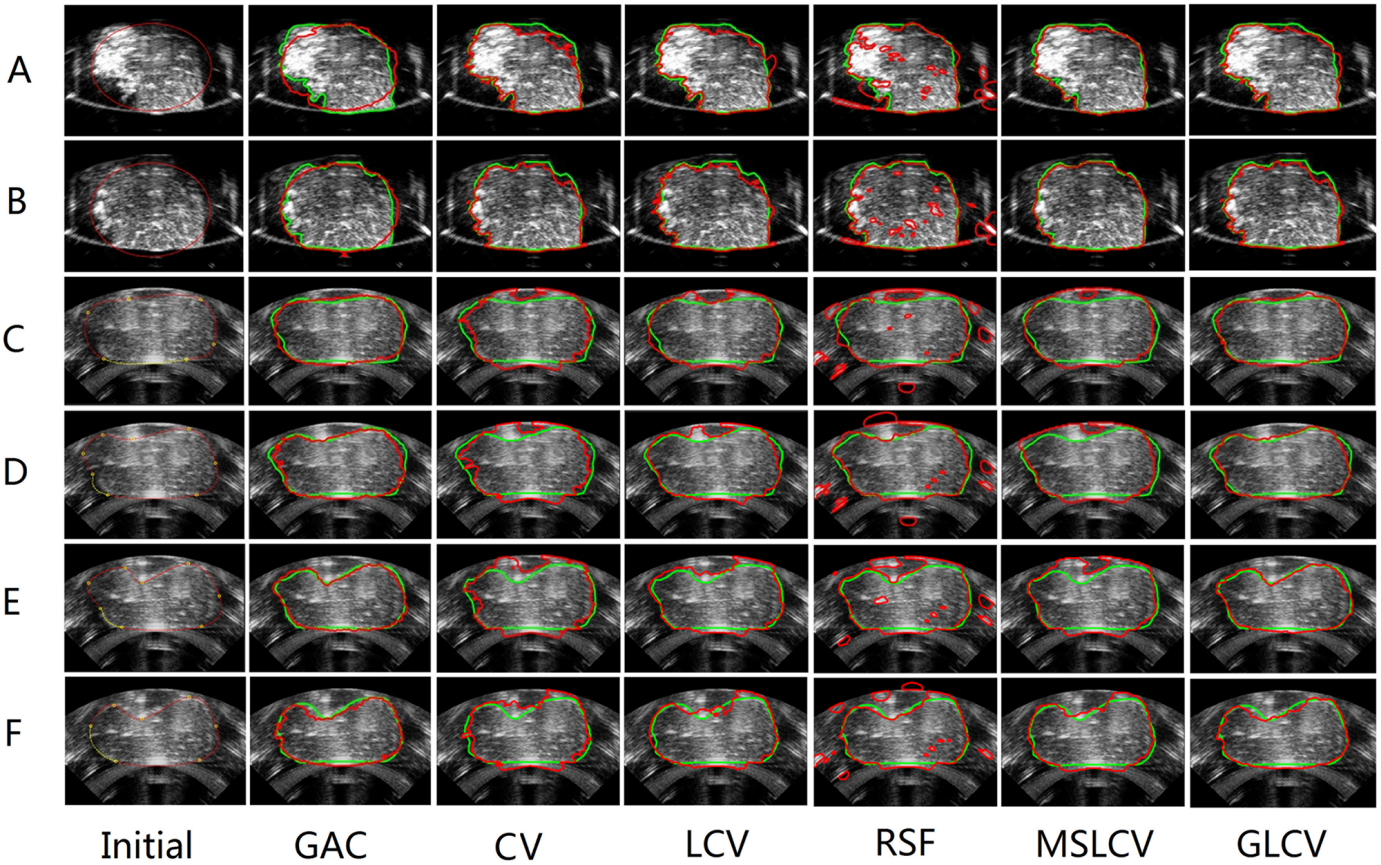
Comparison of the GLCV and five other methods by applying them to segment six HIFU ultrasound images. The first column shows the original images and the initial contours. For images A and B, the initial contour is an ellipse, and for images C, D, E and F, the initial contours are defined by 5–7 connecting points. Columns 2 to 7 show, respectively, the segmentation results for GAC [19], CV [20], LCV [22], RSF [23], MSLCV [24] and GLCV. The green curves are manual segmentation results by an experienced doctor as the ground truth, and the red curves are the final segmentation contours from these methods.

We tested the proposed GLCV model on HIFU ultrasound images of the target tissue and compared our GLCV based method with other well-known methods in segmenting HIFU ultrasound images of the target tissue, including GAC [19], CV [20], LCV [22], RSF [23] and MSLCV [24]. Fig 2 presents the experimental results.

**Fig 3:**
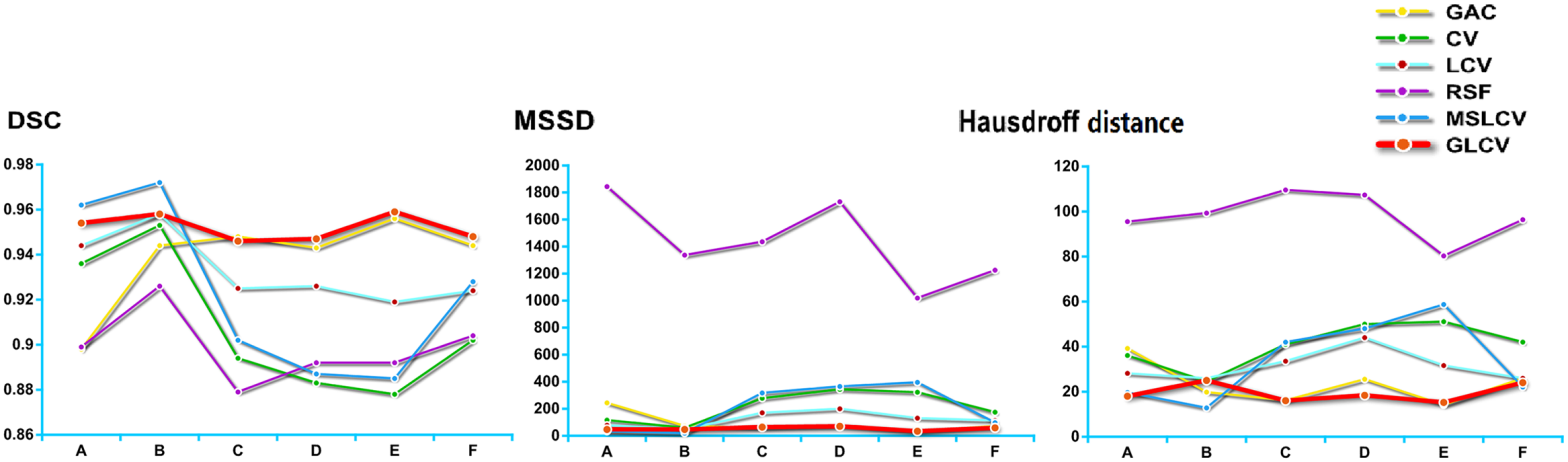
Comparisons of computed DSC [36], MSSD [37] and Hausdroff distance [37] utilizing GAC [19], CV [20], LCV [22], RSF [23], MSLCV [24] and GLCV methods for segmentation of HIFU ultrasound images. The red lines are the performance line of GLCV, indicating the GLCV method achieves the best segmentation performance.

To quantitatively compare the segmentation results of the six methods, we computed the Dice Similarity Coefficient (DSC)[36], Mean Sum of Square Distance (MSSD) [37] and Hausdorff distance [37] with respect to the ground truth. The closer the value of the DSC is to 1, the better the segmentation result. Likewise, the closer the values of the MSSD and Hausdorff distance are to 0, the better the segmentation result. Table 2 shows the values of DSC, MSSD and Hausdorff distance of the six methods depicted in Fig 2. The bold numbers in each row of Table 2 label the best results. It can be observed that the GLCV gains 8 best results, more than gained by GAC, CV, LCV, RSF or MSLCV. Moreover, except for GLCV’s 8 best results, the remaining results of GLCV are almost the second best. The result in Table 2 implies that the GLCV model obtains more accurate and more stable performance than other five methods. Fig 3 shows the comparison of values of DSC, MSSD and Hausdroff distance. Again, the performance of the GLCV over other methods can be observed clearly.

**Table 2:** Comparison of DSC [3 6], MSSD [3 7] and Hausdroff distance [3 7] by utilizing GAC [19], CV [20], LCV [22], RSF [23], MSLCV [24] and GLCV methods for HIFU ultrasound images segmentation.

Images	Standard	GAC	CV	LCV	RSF	MSLCV	GLCV
A	DSC	0.898	0.936	0.944	0.899	**0.962**	0.954
	MSSD	243.63	116.00	78.01	1844.01	**32.94**	46.89
	Hausdorff	39.21	36.07	28.16	95.53	19.65	**18.00**
B	DSC	0.944	0.953	0.958	0.926	**0.972**	0.958
	MSSD	72.56	58.71	48.59	1336.96	**19.11**	46.71
	Hausdorff	19.80	24.52	25.96	99.30	**12.81**	25.00
C	DSC	**0.948**	0.894	0.925	0.879	0.902	0.946
	MSSD	**58.91**	277.26	170.15	1435.74	316.91	64.27
	Hausdorff	16.12	41.00	33.54	109.57	42.00	**16.03**
D	DSC	0.943	0.883	0.926	0.892	0.887	**0.947**
	MSSD	77.09	343.19	199.63	1731.64	365.05	**70.09**
	Hausdorff	25.55	50.00	44.00	107.33	48.00	**18.39**
E	DSC	0.956	0.878	0.919	0.892	0.885	**0.959**
	MSSD	**33.75**	322.24	130.28	1018.78	395.61	33.77
	Hausdorff	**14.14**	51.09	31.62	80.28	58.73	15.23
F	DSC	0.944	0.902	0.924	0.904	0.928	**0.948**
	MSSD	72.56	174.67	113.09	1225.40	96.82	**58.75**
	Hausdorff	25.94	42.01	25.94	96.38	**22.09**	24.04

The bold numbers in each row label the best results. It can be observed that GLCV has gained 8 best results, which is more than GAC, CV, LCV, RSF and MSLCV. Moreover, except for GLCV’s 8 best results, the remaining results of GLCV are almost the second best results. The results imply that GLCV model obtains more accurate and more stable performance than other five methods.

### 3.2 Modeling, Prediction and Validation Results

Based on the versatile particle framework, we established particle models representing target tissue, fluids, and rigid body and adopted the multiphase coupling method to compute the coupling between them. To experimentally validate our method, we applied it to the target tissue, which allowed us to both train our parameters and verify our experimental results based on both contour and DSC values.

The ultrasound-guided HIFU equipment was used in our experiment to record time series of ultrasound images with mechanical movement and deformation. A single cross section of the target tissue was recorded while an external force was applied. Starting from the 20^th^ frame, 18 HIFU ultrasound images of the selected cross section in every other 10 frames were used as the ground truth images, which were segmented manually by an experienced physician to obtain ground truth contours. We utilized 1^st^~9^th^ ground truth contours as the training set, and the 10^th^~18^th^ ground truth contours as the validation set. Using the versatile particle framework, we modeled the environment surrounding the target tissue. Starting from the 20^th^ time steps, we output the calculation contours every other 10 time steps. We adopted the iterative parameter estimation algorithm in Table 1 to estimate the essential parameters of the versatile particle framework by fitting our 1^st^~9^th^ output contours within the deviation compared with that of the 1^st^~9^th^ ground truth contours. In the experiment, the particle number of target tissue, fluids and rigid body were, respectively 4399, 3344 and 120. The acquired essential parameters of the versatile particle framework are shown in Table 3. We adopted the DSC values as the comparison standard to evaluate the calculation accuracy. The DSC was selected because it is a relative value indicating the closeness of prediction between 0 (worst) and 1 (best). The DSC values of the training set and validation set are shown in Table 4. It can be seen that the acquired essential parameters can well fit the training set and the prediction results are very close to the validation set.

**Table 3:** Essential Parameters of the Versatile Particle Framework.

Parameters	Value	Unit
Young modulus *E*	13	*KPa*
Poisson’s ratio *η*	0.47	-
Smooth length *h*	2×10^-5^	*m*
Elastic tissue Particle’s mass *m*	3×10^-5^	*kg*
External Force *F*	2.5×10^-1^	*N*
*β*_1_	1	-
*Β*_2_	0.5	-

**Table 4:** DSC Values of Training Set and Validation Set.

**Training set (frame number)**	20^th^	30^th^	40^th^	50^th^	60^th^	70^th^	80^th^	90^th^	100^th^	**average**
DSC	0.9654	0.9590	0.9662	0.9683	0.9725	0.9712	0.9752	0.9634	0.9711	**0.9680**
**Validation set (frame number)**	110^th^	120^th^	130^th^	140^th^	150^th^	160^th^	170^th^	180^th^	190^th^	**average**
DSC	0.9752	0.9634	0.9770	0.9711	0.9752	0.9634	0.9770	0.9639	0.9615	**0.9697**

The experimental results indicates the acquired essential parameters can well fit the training set and the prediction results is very close to the validation set.

**Fig 4:**
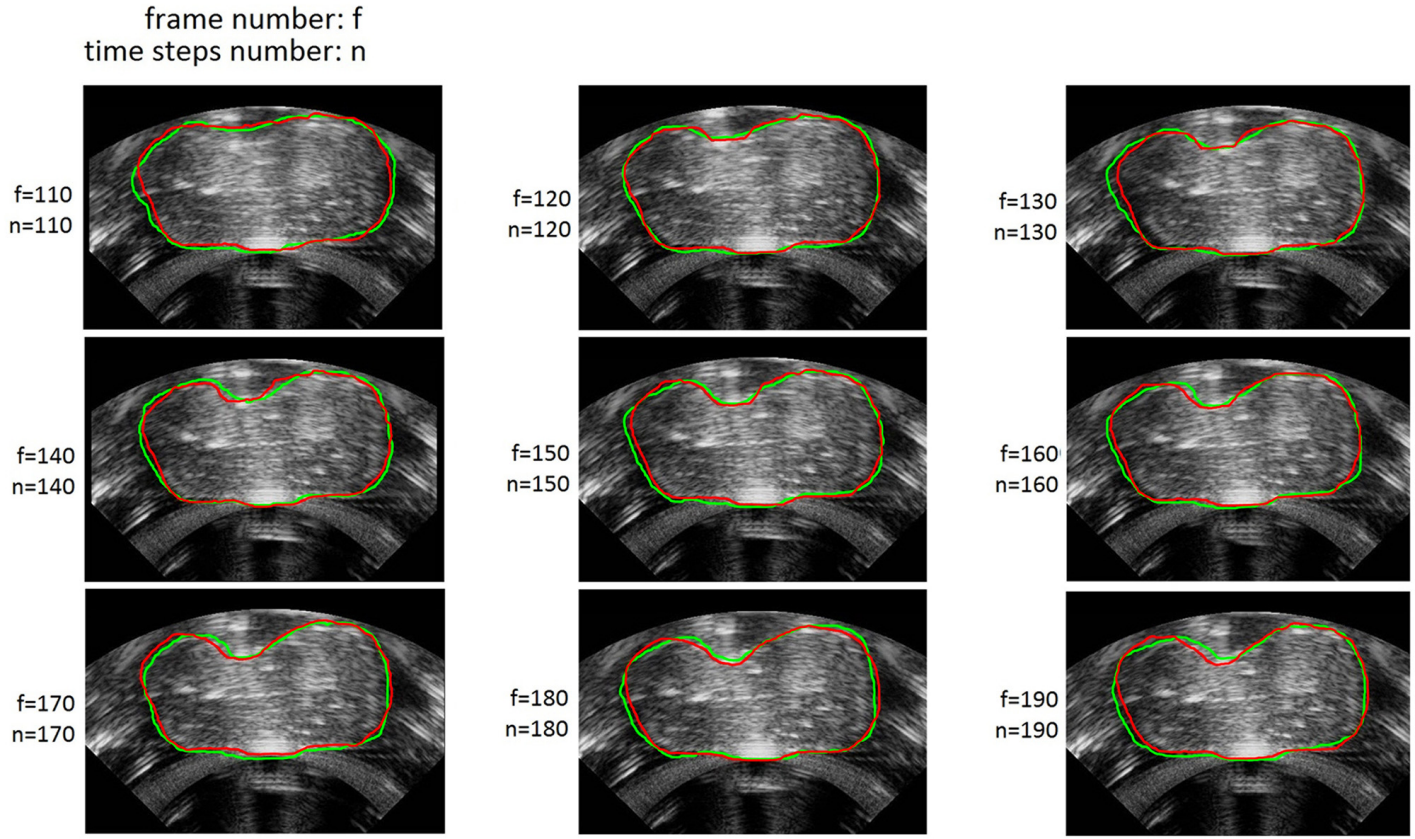
Comparison of ground truth contours (green contour) with the corresponding predicted contours (red contour). The figures are from the validation set. The results illustrate that the predicted contours are very close to the ground truth contours, indicating the effectiveness of our method.

The ground truth contours (green) and predicted contour (red) are shown in Fig 4, which illustrates the predicted contours are very close to the ground truth contours. Meanwhile, the lowest, highest and average DSC values of predicted contours and the ground truth contours are respectively 0.9615, 0.9770 and 0.9697, which suggests that our method can effectively predict the dynamic contours of the target tissue as it moves and deforms during ultrasound-guided HIFU therapy.

## Discussion

Our method can predict the movement and deformation of the target tissue by analyzing the recorded images of dynamic changes in response to external forces. It provides the opportunity to adjust in a timely manner the focal region of ultrasound-guided HIFU equipment, allowing the HIFU treatment head to remain aligned with the tumorigenic tissue throughout the course of therapy. Compared with the Kalman filtering method [38], which also can be used as moving object’s detection and can quickly obtain the moving object’s approximate positional information represented by a tracking window indicating the object’s position, our method can obtain the accurate contour (red curve in Fig 4) of the target tissue rather than an approximate tracking window, which is essential for the adjustment of the HIFU treatment head.

However, there exist challenges and limitations. First, while the proposed GLCV method can accurately segment a single target tissue, it is still difficult to segment target tissue’s HIFU ultrasound images which are extremely non-homogeneous as the characteristic function *t*(*x*) cannot measure the grey level uniformity around each point perfectly. Second, the GLCV model inherits the drawback of the LCV model that the contour evolution process increases the amount of calculation. The average time for segmenting a HIFU ultrasound image is 15.1 seconds. However, segmentation by GLCV is still faster than manually segmenting the HIFU ultrasound images and some segmentation task can be accomplished in pre-computing process before prediction. Third, the performance of the proposed versatile particle framework depends on the number of particles. If the number is too large, the real-time capability of the model is affected. In our experiment, we achieved a frame rate of 11.5 frames/sec which is close to real-time computation. For more complex scenarios with a large number of particles, it could be a challenge to obtain real-time prediction results.

Despite these limitations, this paper has proposed a new method to segment HIFU ultrasound images instead of manually segmenting and predicting the movement and deformation of target tissue during ultrasound-guided HIFU therapy to make adjustment to the HIFU treatment head, and the correctness of our method has been validated by the experimental results.

The implementation of our method *in vivo* is similar to the implementation process shown in Fig 1. After the motion of target tissue starts, it should be monitored in real-time. Meanwhile, acquiring essential parameters and predicting target tissue’s motion should be carried out consecutively and repetitively. The GLCV based segmentation method can be used directly in HIFU therapy for the segmentation of tumors. However, *in vivo*, the motion of target tissue is more complex than that in our experiments. To implement our tissue motion prediction method *in vivo*, in our future work we will study more complex forms of external forces and accelerate the computation to enhance real-time performance with a high prediction accuracy.

## Conclusions

We have presented a novel method to model and predict target tissue movement and deformation during ultrasound-guided HIFU therapy. We first proposed the GLCV model, which utilizes edge information and adopts a localized active contour model to segment HIFU images effectively. The segmentation results are then used to reconstruct a 3D model of the target tissue. To predict the movement and deformation that the target tissue undergoes when exposed to the external force, we propose a versatile particle framework to compute multiphase coupling among target tissue, rigid body and fluids. After estimating essential parameters of the versatile particle framework using the iterative parameter estimation algorithm, we utilize these parameters in the versatile particle framework to predict the movement and deformation of the target tissue. Our experimental results show that the lowest, highest and average DSC values of predicted contours and the ground truth contours are 0.9615, 0.9770 and 0.9697, respectively. Our experimental results also suggest that our method can predict the movement and deformation of target tissue effectively.
